# The estrogen–ferroptosis axis in postmenopausal osteoporosis, osteoarthritis, and intervertebral disc degeneration: shared mechanisms and emerging evidence

**DOI:** 10.3389/fimmu.2026.1951300

**Published:** 2026-08-26

**Authors:** Kaiyuan Zheng, Peiyue Zhang, Siyu Wang, Li Wang, Xiaoyan Zheng, Nik Nasihah Nik Ramli, Juan Tan

**Affiliations:** 1 Department of Rehabilitation Medicine, Department of Nursing, Intensive Care Unit, Department of Ultrasound Medicine, Affiliated Hospital of North Sichuan Medical College, Nanchong, Sichuan, China; 2 School of Graduate Studies, Post Graduate Centre, Neuroscience & Mental Well-being Centre (NeuroMIND), International Medical School, Management & Science University, Shah Alam, Selangor, Malaysia; 3 School of Nursing, North Sichuan Medical College, Nanchong, Sichuan, China

**Keywords:** estrogen deficiency, ferroptosis, intervertebral disc degeneration, musculoskeletal degeneration, osteoarthritis, postmenopausal osteoporosis

## Abstract

Estrogen deficiency is a major risk factor for degenerative disorders of the musculoskeletal system in postmenopausal women, while ferroptosis, an iron-dependent form of programmed cell death, has been implicated in the pathogenesis of various osteoarticular diseases. Recent studies indicate an interplay between estrogen signaling and ferroptosis, with evidence relatively well supported in postmenopausal osteoporosis (PMOP), emerging in osteoarthritis (OA), and still limited in intervertebral disc degeneration (IVDD). Estrogen deficiency may disrupt iron homeostasis, lipid metabolism, and antioxidant defense systems, thereby increasing ferroptosis susceptibility across musculoskeletal tissues. This review summarizes the roles of estrogen and recent research progress in these three common degenerative diseases from the perspective of ferroptosis-related mechanisms. Particular emphasis is placed on the shared mechanisms and tissue-specific manifestations of this regulatory axis across these diseases. Finally, we highlight therapeutic opportunities and translational challenges associated with targeting this axis, including estrogen receptor subtype modulation, ferroptosis inhibition, and local delivery strategies. Overall, the estrogen–ferroptosis axis may provide a mechanistic framework for understanding postmenopausal musculoskeletal comorbidity and developing targeted interventions for degenerative musculoskeletal disorders.

## 1 Introduction

Estrogen plays a pivotal role in preserving the homeostasis of the skeletal and joint systems, and its decline is widely recognized as a major contributor to the initiation and progression of degenerative disorders such as postmenopausal osteoporosis (PMOP), osteoarthritis (OA), and intervertebral disc degeneration (IVDD) (1, 2). A substantial body of evidence has shown that estrogen exerts protective effects across multiple musculoskeletal tissues. In bone, it is essential for maintaining the balance of bone remodeling (3). In articular cartilage, estrogen attenuates cartilage degeneration by suppressing inflammatory responses and the expression of matrix-degrading enzymes (4). In the intervertebral disc, it is thought to preserve the functional stability of nucleus pulposus cells through the regulation of apoptosis, oxidative stress, and extracellular matrix metabolism (5). Notably, epidemiological and clinical observations suggest that although PMOP, OA, and IVDD arise in distinct anatomical structures and present with different clinical manifestations, they often coexist and tend to progress more rapidly under estrogen-deficient conditions (2, 6). To date, studies on estrogen have mainly focused on inflammation, hormone signaling, and apoptosis. However, whether these disorders share additional cell-death mechanisms that are sensitive to estrogen deficiency remains incompletely understood (7, 8).

In recent years, ferroptosis, a form of regulated cell death characterized by iron-dependent lipid peroxidation, has attracted increasing attention as a mechanistic link between metabolic imbalance, oxidative damage, and tissue degeneration (9). At its core, ferroptosis involves the disruption of several critical processes, including intracellular iron homeostasis, lipid redox balance, and the System Xc^−^/GSH/GPX4 antioxidant defense axis. Growing evidence indicates that ferroptosis is actively involved in the development and progression of multiple degenerative bone and joint disorders. In osteoporosis, factors such as hyperglycemia, aging, and oxidative stress can trigger ferroptosis in osteoblasts, often accompanied by impaired antioxidant capacity and mitochondrial dysfunction (10, 11). In OA, inflammatory stimuli such as IL-1β promote the accumulation of reactive oxygen species and lipid peroxides while suppressing glutathione peroxidase 4 (GPX4) expression, thereby inducing chondrocyte ferroptosis and accelerating extracellular matrix degradation (12). In IVDD, ferroptosis in nucleus pulposus cells has been closely associated with heightened oxidative stress, impaired extracellular matrix synthesis, and progressive disc degeneration (13). Collectively, these findings suggest that ferroptosis may represent a key common execution pathway across degenerative disorders affecting distinct musculoskeletal tissues.

Recent mechanistic studies have established estrogen as an important determinant of cellular ferroptosis susceptibility (14). Conversely, disruption of estrogen/ESR1 signaling or estrogen-deprived conditions can weaken anti-ferroptotic defenses and increase cellular susceptibility to ferroptosis (15). Beyond the classical estrogen receptor α/β (ERα/ERβ)-mediated pathways, emerging evidence suggests that the G protein-coupled estrogen receptor (GPER/GPR30) also participates in the regulation of ferroptosis, for example by modulating YAP1 phosphorylation and ferritin heavy chain 1 (FTH1) expression (16). At the mechanistic level, estrogen may regulate ferroptosis through several interconnected processes, including activation of Nrf2-dependent antioxidant defenses (17), maintenance of the SLC7A11/GSH/GPX4 axis (18), regulation of intracellular iron homeostasis through proteins such as FTH1 (16), and modulation of lipid metabolism and lipid peroxidation (14, 15). Collectively, these findings support a direct regulatory relationship between estrogen signaling and ferroptosis and provide a mechanistic basis for the potential involvement of this axis in degenerative musculoskeletal diseases.

Although current studies have separately implicated ferroptosis in PMOP, OA, and IVDD and suggested a protective role for estrogen, the estrogen–ferroptosis relationship has not yet been systematically integrated across these disorders. In particular, the extent to which this axis is shared across different musculoskeletal cell types, and the involvement of ferroptosis-related mechanisms beyond the canonical Nrf2/GPX4 pathway remain unclear. These gaps limit a comprehensive understanding of its role in degenerative musculoskeletal diseases. Accordingly, this review systematically evaluates the estrogen–ferroptosis regulatory axis in PMOP, OA, and IVDD, while distinguishing direct experimental evidence from indirect mechanistic inference. By comparing the current evidence across these disorders, we aim to identify common mechanistic features, clarify disease-specific differences and evidence gaps, and discuss the potential therapeutic implications of this regulatory axis.

## 2 Molecular mechanisms linking estrogen signaling to ferroptosis regulation

As a pivotal sex hormone, the biological effects of estrogen are primarily mediated by two classes of receptors: the classical nuclear receptors, ERα and ERβ, and the membrane receptor, GPER. Among these receptors, ERα and ERβ mainly mediate genomic effects. Upon ligand binding, these receptors undergo conformational rearrangement, form dimers, and translocate into the nucleus, where they bind to estrogen response elements within the promoter regions of target genes, thereby regulating the transcription of a broad range of genes involved in cell proliferation, differentiation, and redox homeostasis (15, 19). Conversely, GPER mediates more rapid non-genomic signaling responses, which can promptly alter cellular stress states and survival programs through pathways such as PI3K/AKT, ERK, cAMP/PKA, and Ca²^+^ signaling (20). This dual mode of action, combining nuclear receptor-mediated transcriptional regulation with membrane receptor-mediated rapid signal transduction, enables estrogen to coordinate multiple cellular functions at the upstream level, including the remodeling of ferroptosis susceptibility.

**Figure 1:**
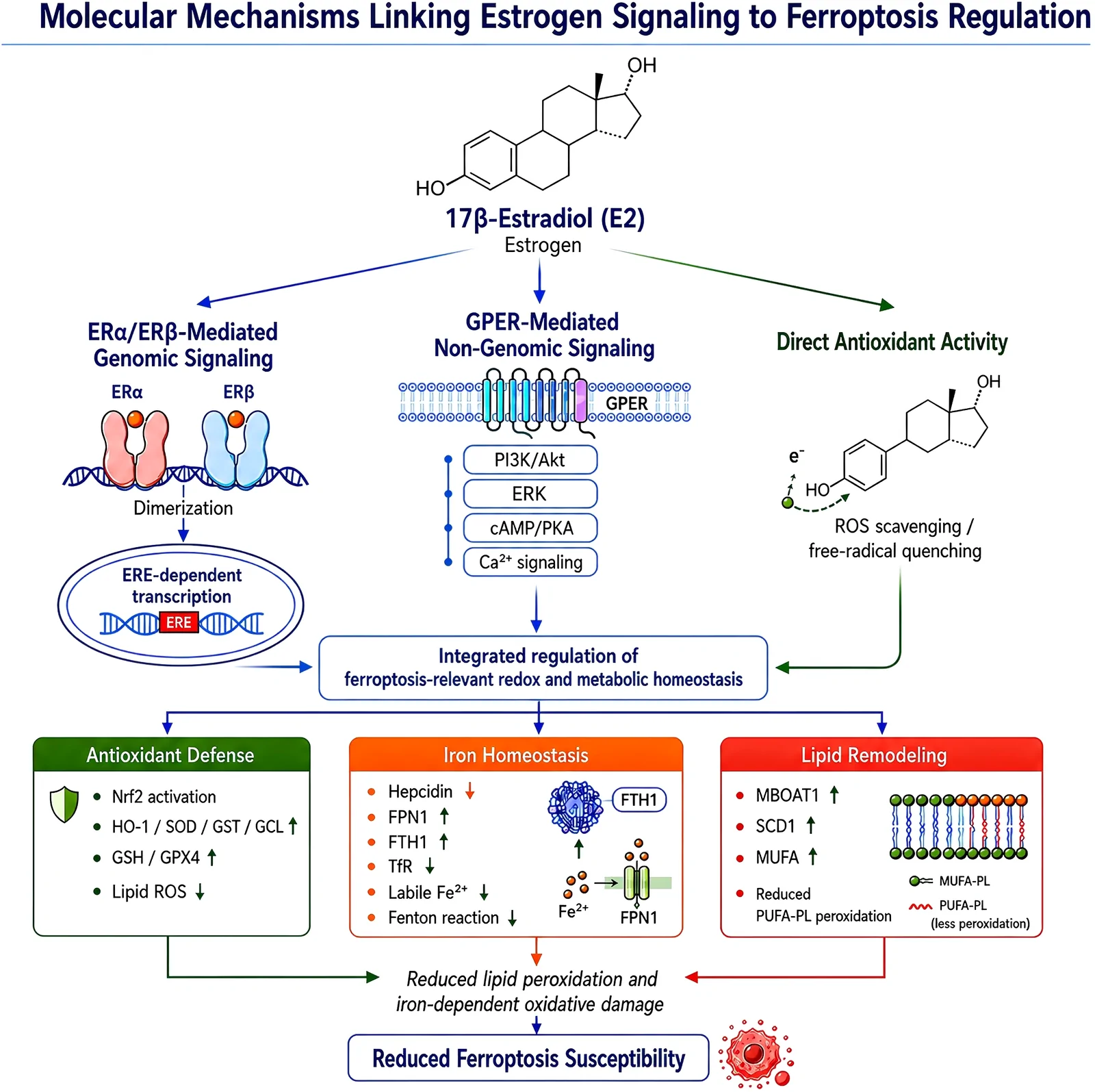
Molecular mechanisms linking estrogen signaling to ferroptosis regulation. Estrogen modulates ferroptosis through ERα/ERβ-mediated genomic signaling, GPER-mediated non-genomic signaling, and receptor-independent direct antioxidant activity. These pathways converge on the regulation of antioxidant defense, iron homeostasis, and lipid remodeling, collectively limiting lipid peroxidation and iron-dependent oxidative damage and thereby reducing ferroptosis susceptibility.

Importantly, the influence of estrogen on ferroptosis is not entirely restricted to receptor-mediated signaling. Its unique phenolic structure, particularly the free phenolic hydroxyl group on the A ring, is considered one of the chemical bases underlying its direct antioxidant activity. This structural feature allows estrogen to scavenge free radicals and suppress lipid peroxidation, thereby alleviating oxidative stress-mediated cellular damage to some extent (21, 22). Therefore, mechanistically, estrogen does not merely target a single ferroptosis-related molecule; rather, it is more likely to exert broad regulatory effects on the initiation and progression of ferroptosis by modulating cellular redox status, lipid peroxidation, and the iron homeostasis-associated microenvironment (**Figure 1**).

### 2.1 Estrogen suppresses ferroptosis by enhancing antioxidant defense

Uncontrolled lipid peroxidation is the executional core of ferroptosis. Accordingly, the preservation of cellular antioxidant defense represents a primary mechanism by which estrogen suppresses ferroptosis. Early studies demonstrated that 17β-estradiol reduces reactive oxygen species (ROS) generation in vascular cells and upregulates the expression and activity of manganese superoxide dismutase (MnSOD) and extracellular superoxide dismutase (ecSOD), thereby attenuating oxidative stress (23). In addition, estrogen enhances glutathione-dependent reducing systems. Studies have shown that estrogen can activate the Nrf2-dependent antioxidant transcriptional program, promoting the expression of antioxidant and detoxification-related molecules, including heme oxygenase-1 (HO-1), superoxide dismutase (SOD), glutathione S-transferase (GST), and glutamate-cysteine ligase (GCL), thereby strengthening the cellular capacity to eliminate free radicals and maintain glutathione homeostasis (24, 25).

Given that the Nrf2–GPX4 axis constitutes a central defense system for detoxifying lipid peroxides and preserving membrane redox homeostasis, activation of this pathway is directly relevant to the suppression of ferroptosis. Recent studies have further demonstrated that estrogen deficiency accelerates lipid peroxidation and iron deposition through inhibition of the Nrf2/GPX4 pathway, thereby triggering ferroptosis in endothelial cells and promoting the progression of postmenopausal atherosclerosis (17). In summary, these findings suggest that estrogen inhibits ferroptosis by reinforcing cellular antioxidant defenses and preserving the function of the Nrf2/GPX4 protective axis, ultimately limiting lipid peroxide accumulation and reducing the likelihood of ferroptotic cell death.

### 2.2 Estrogen reduces ferroptosis susceptibility by remodeling iron homeostasis and lipid metabolism

Beyond strengthening antioxidant defenses, estrogen may also reduce ferroptosis by remodeling iron homeostasis and lipid metabolism. The occurrence of ferroptosis depends on two key conditions: first, expansion of the intracellular labile iron pool, which facilitates Fenton chemistry; and second, enrichment of membrane phospholipids containing polyunsaturated fatty acids (PUFAs), which are highly prone to peroxidation. Therefore, any mechanism capable of limiting free iron accumulation, promoting iron efflux, or reducing the availability of peroxidation-susceptible lipid substrates may suppress ferroptosis at an upstream level.

Available evidence indicates that 17β-estradiol suppresses the transcription of hepcidin through an estrogen response element-dependent mechanism, thereby favoring the maintenance of ferroportin (FPN1) and cellular iron export (26). At the cellular level, estrogen can also affect the expression of multiple iron metabolism-related molecules, including ferroportin, transferrin receptor (TfR), and ferritin (FTH1), thereby altering the cellular states of iron uptake, storage, and export (27). Mechanistically, such regulation helps limit the accumulation of pro-oxidant Fe²^+^ and reduces the probability of triggering lipid peroxidation chain reactions. In other words, estrogen does not merely passively buffer oxidative damage after ferroptosis has been initiated; rather, it lowers the iron-dependent basis required for ferroptosis by reshaping intracellular iron trafficking at its source.

On the other hand, the enrichment of PUFA-containing phospholipids in cellular membranes and their subsequent peroxidation are closely associated with ferroptosis. Recent studies have shown that MBOAT1, a membrane phospholipid remodeling enzyme, can be transcriptionally upregulated by ER and suppress ferroptosis by reshaping cellular phospholipid composition, an effect that appears to be at least partially independent of the GPX4/FSP1 system (15). In another study, 17β-estradiol was found to induce the expression and activity of the lipid metabolic enzyme stearoyl-CoA desaturase 1 (SCD1) in estrogen receptor-positive cells, thereby increasing the intracellular ratio of monounsaturated fatty acids (MUFAs) to saturated fatty acids (SFAs) (28). Since MUFA enrichment is known to inhibit membrane lipid peroxidation, this finding suggests that estrogen may enhance cellular resistance to ferroptosis by promoting fatty acid desaturation and remodeling membrane lipid composition. Taken together, these studies indicate that the regulatory effects of estrogen on membrane lipid composition and fatty acid metabolism may constitute another important mechanism through which estrogen modulates ferroptosis (**Table 1**).

**Table 1:** Molecular mechanisms linking estrogen signaling to ferroptosis regulation.

Estrogen action mode	Signaling or regulatory context	Main mechanistic implication	Ferroptosis-related effect	Representative molecules
ERα/ERβ-mediated genomic signaling	Ligand-bound ER activation and transcriptional regulation	Modulates genes involved in redox balance, antioxidant defense, and cellular stress adaptation	Reduced lipid peroxide accumulation	Nrf2, GPX4, GSH, HO-1, GCL
	ER-associated regulation of iron metabolism	May influence iron uptake, storage, and export, thereby limiting intracellular pro-oxidant iron availability	Reduced labile iron burden	Hepcidin, FPN1, TfR, FTH1
	Regulation of phospholipid remodeling and fatty acid metabolism	Alters membrane lipid composition and reduces susceptibility to lipid peroxidation	Reduced PUFA-phospholipid peroxidation susceptibility	MBOAT1, SCD1
GPER-mediated non-genomic signaling	Rapid membrane-initiated signal transduction	Activates pro-survival and stress-response pathways that may affect ferroptosis sensitivity	Reduced ferroptosis susceptibility	GPER, PI3K/AKT, ERK, cAMP/PKA, Ca²^+^
Receptor-independent antioxidant effect	Direct chemical antioxidant activity of estrogen	Phenolic hydroxyl group can scavenge radicals and suppress lipid peroxidation	Reduced ferroptosis initiation	17β-estradiol

## 3 Role of the estrogen–ferroptosis axis in PMOP, OA, and IVDD

PMOP, OA, and IVDD frequently overlap in aging women after estrogen decline, reflecting shared features such as oxidative stress, iron dyshomeostasis, inflammation, and progressive extracellular matrix or skeletal tissue deterioration.

### 3.1 Role of the estrogen–ferroptosis axis in PMOP

**Figure 2:**
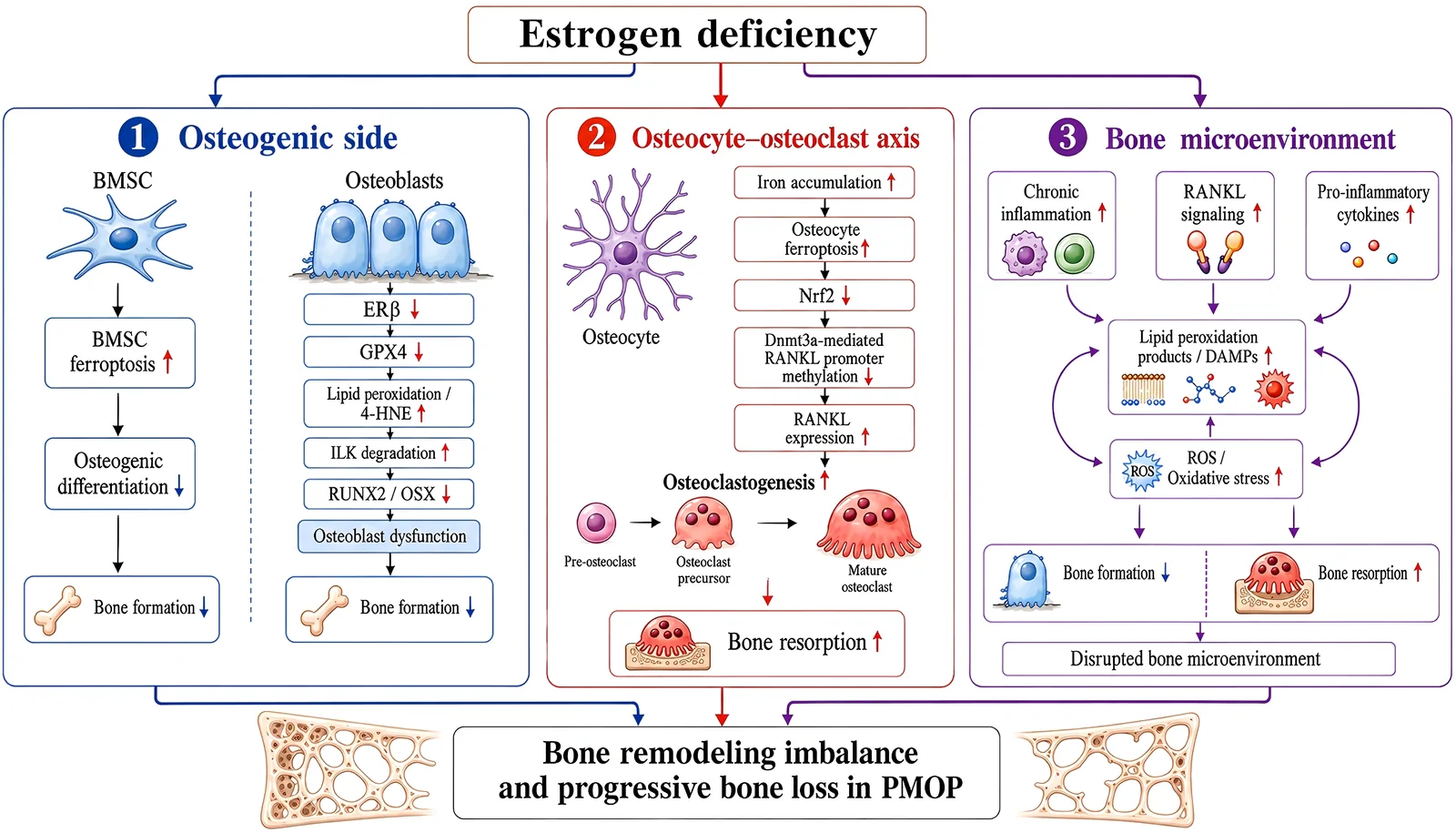
Role of the estrogen–ferroptosis axis in bone remodeling imbalance in PMOP. Estrogen deficiency increases ferroptotic susceptibility in bone cells. Enhanced ferroptosis in BMSCs and osteoblasts impairs osteogenic differentiation and function, leading to reduced bone formation, whereas osteocyte ferroptosis promotes RANKL-mediated osteoclastogenesis and increases bone resorption. Meanwhile, ferroptosis-associated lipid peroxidation, ROS, DAMPs, and inflammatory signaling further disrupt the bone microenvironment, collectively amplifying bone remodeling imbalance and progressive bone loss in PMOP.

PMOP is fundamentally characterized by an imbalance in bone remodeling coupling under conditions of estrogen deficiency. This imbalance manifests as reduced bone formation, enhanced bone resorption, and disruption of bone microenvironment homeostasis. In recent years, ferroptosis has been recognized as a critical nexus linking estrogen decline, increased oxidative stress, iron metabolism dysregulation, and dysfunction of bone cells (29). Emerging evidence suggests that estrogen regulates bone metabolism not only through classical endocrine mechanisms but also by modulating iron homeostasis pathways, thereby influencing the tolerance of bone tissue to lipid peroxidative damage (30) (**Figure 2**).

#### 3.1.1 Estrogen deficiency-induced ferroptosis in osteoblasts and BMSCs

Within the osteogenic lineage, osteoblasts represent the cell type with the most direct mechanistic evidence linking estrogen deficiency to ferroptosis. Estrogen maintains osteoblast ferroptosis resistance by promoting GPX4 expression through ERβ, whereas estrogen deficiency disrupts this axis, leading to impaired detoxification of phospholipid peroxides, increased lipid peroxidation, and accumulation of 4-hydroxynonenal (4-HNE). The elevated 4-HNE promotes integrin-linked kinase (ILK) degradation, suppresses RUNX2/OSX-mediated osteogenic differentiation, and ultimately impairs bone formation. Consistently, osteoblast-specific Gpx4 deficiency or GPX4 inhibition exacerbates ferroptotic damage and bone loss, while ferroptosis inhibition or GPX4 activation restores osteogenic activity (30). In addition to ERβ–GPX4 regulation, other mechanisms may contribute to osteoblast ferroptosis in osteoporotic conditions. Increased DNMT activity promotes GPX4 promoter hypermethylation and suppresses GPX4 expression in OVX models and osteoporotic bone, whereas inhibition of DNMTs alleviates ferroptosis and bone loss (31). Moreover, activation of the CEBPA/ALOX15B axis enhances lipid peroxidation and osteoblast ferroptosis, contributing to impaired osteogenesis in PMOP (32). Pharmacological evidence further supports the pathological relevance of osteoblast ferroptosis, as vitamin K2 attenuates bone loss in OVX mice by suppressing PGE2-mediated ferroptosis through activation of Nrf2 and upregulation of CBR1 (33). Although the direct regulation of these pathways by estrogen requires further investigation, current evidence indicates that estrogen deficiency promotes osteoblast ferroptosis through convergent mechanisms involving reduced antioxidant defense and enhanced lipid-peroxidation signaling.

Evidence from BMSCs further indicates that ferroptosis affects not only mature osteoblasts but also the cellular source of bone-forming cells. Studies have shown that 4-octyl itaconate (4-OI) activates the Nrf2 pathway in an erastin-induced model of BMSC ferroptosis, reduces ROS production and lipid peroxidation, restores GPX4 expression, and enhances osteogenic differentiation. In ovariectomized (OVX) mice, 4-OI likewise ameliorates bone loss and upregulates Nrf2/GPX4 signaling in bone tissue (34). Notably, estrogen-related signaling may also influence ferroptosis in BMSCs through the membrane-associated estrogen receptor GPER. A 2025 study reported that activation of GPER promotes iron export through the BMP-6/HEP/FPN signaling axis, thereby inhibiting ferroptosis in BMSCs and enhancing osteogenic differentiation (35). In addition, studies have shown that suppressing ferroptosis in bone formation–related cells exerts a clear osteoprotective effect in models of postmenopausal osteoporosis. For example, sarsasapogenin inhibits BMSC ferroptosis in an OVX model by stabilizing GPX4 and activating the GPX4/SLIT3/ROBO1 axis, restoring osteogenesis–angiogenesis coupling and thereby improving bone loss caused by estrogen deficiency (36). Together, these findings support a role for BMSC ferroptosis in impaired bone formation under estrogen-deficient conditions, although the molecular mechanisms directly linking estrogen withdrawal to ferroptosis in BMSCs remain less well defined than those in osteoblasts.

#### 3.1.2 Estrogen signaling and ferroptosis-related regulation of osteoclastogenesis

Estrogen is a major physiological regulator of osteoclastogenesis and bone resorption. 17β-Estradiol suppresses RANKL-induced osteoclast differentiation through estrogen receptor-dependent mechanisms and regulates mitochondrial metabolism and survival during osteoclast formation (37, 38). Although ferroptosis-related processes have also been implicated in osteoclast differentiation and may exert stage- or stress-dependent effects (39, 40), direct evidence that estrogen signaling regulates ferroptosis specifically within osteoclasts remains limited.

However, indirect evidence suggests that estrogen deficiency may promote osteoclastogenesis through ferroptosis-mediated osteocyte–osteoclast crosstalk. Jiang et al. showed that estrogen withdrawal promotes iron accumulation and ferroptosis in osteocytes, accompanied by increased RANKL expression (41). In direct osteocyte–osteoclast coculture, ferroptotic osteocytes enhanced osteoclast formation and bone-resorption activity, whereas ferroptosis inhibition attenuated these effects. Mechanistically, osteocytic ferroptosis suppressed Nrf2 and reduced Dnmt3a-mediated methylation of the RANKL promoter, thereby increasing RANKL expression and promoting osteoclastogenesis. Consistently, osteocyte-specific GPX4 deficiency aggravated osteoclast activation and bone loss in ovariectomized mice. These findings support an indirect “estrogen deficiency–osteocyte ferroptosis–Nrf2/Dnmt3a/RANKL–osteoclastogenesis” pathway and suggest that ferroptosis-mediated intercellular crosstalk may contribute to enhanced bone resorption in PMOP. However, whether estrogen directly modulates ferroptotic susceptibility in osteoclasts remains to be determined.

#### 3.1.3 Estrogen deficiency, inflammation, and ferroptosis: a microenvironmental link to bone remodeling imbalance

PMOP is not caused by abnormalities in a single bone cell population, but rather by a broader imbalance of the bone microenvironment. Estrogen deficiency can induce low-grade chronic inflammation, promote the release of inflammatory cytokines, and enhance RANKL signaling (42). Meanwhile, lipid peroxidation products and damage-associated molecules generated during ferroptosis may further amplify inflammatory responses, forming a vicious cycle of “inflammation–lipid peroxidation–bone remodeling imbalance” (29). In this process, injury to osteoblasts and BMSCs reduces bone formation, whereas enhanced osteoclast activation increases bone resorption. Together, these findings support a broader view of the estrogen–ferroptosis axis in PMOP as a coordinated network involving bone-forming cells, osteoclasts, and the osteoimmune microenvironment.

### 3.2 The role of the estrogen–ferroptosis axis in OA

OA is a whole-joint disease involving articular cartilage, the synovium, subchondral bone, and the local immune microenvironment. Its core pathological features include chondrocyte death, extracellular matrix (ECM) degradation, synovial inflammation, and abnormal subchondral bone remodeling. In recent years, ferroptosis, characterized by iron-dependent lipid peroxidation, has been recognized as an important mechanism linking chondrocyte injury, matrix degradation, synovial inflammation, and joint structural damage (43). Meanwhile, epidemiological evidence suggests an association between postmenopausal estrogen decline and the onset and progression of OA, indicating that estrogen deficiency may promote disease progression by increasing the susceptibility of joint tissues to ferroptosis (44).

#### 3.2.1 Estrogen-mediated regulation of chondrocyte ferroptosis

Chondrocytes are the only resident cells in articular cartilage, and their survival status directly determines the balance between ECM synthesis and degradation. Under physiological conditions, chondrocytes maintain cartilage matrix homeostasis by synthesizing type II collagen (COL2A1) and aggrecan. In OA cartilage, Fe²^+^ accumulation, enhanced lipid peroxidation, mitochondrial damage, and impaired GPX4 function have been observed in chondrocytes. Moreover, the ferroptosis inhibitor ferrostatin-1 can partially reverse chondrocyte injury and ECM degradation, suggesting that ferroptosis is involved in OA cartilage degeneration (45).

Regarding estrogen receptor-mediated regulation of chondrocyte ferroptosis, the most direct evidence currently comes from studies on GPER. It has been shown that GPER expression is decreased in OA cartilage tissues, whereas the GPER agonist G1 improves locomotor function and alleviates joint pathological damage in OA mice. In an erastin-induced *in vitro* model of chondrocyte ferroptosis, G1 increases cell viability, reduces Fe²^+^, ROS, MDA, and lipid peroxidation levels, restores mitochondrial membrane potential, and decreases the labile iron burden by inhibiting YAP1 phosphorylation, enhancing YAP1 activation, and upregulating FTH1 expression, thereby suppressing chondrocyte ferroptosis (16). In addition, 17β-estradiol can enhance chondrocyte viability, inhibit excessive mitophagy, and alleviate oxidative injury through GPER- and PI3K/Akt-mTOR-related pathways (46). These findings suggest that the GPER–YAP1–FTH1 axis may represent a key mechanism through which estrogen signaling regulates iron homeostasis and ferroptosis susceptibility in OA chondrocytes.

In addition to GPER, the classical estrogen receptors ERα and ERβ may also participate in the maintenance of cartilage homeostasis. Previous studies have detected the expression of ERα and ERβ in human articular chondrocytes, indicating that chondrocytes possess the molecular basis for responding to estrogen signaling. Estrogen signaling mediated by these receptors may help maintain chondrocyte proliferation and phenotypic stability, promote the synthesis of ECM components, and enhance antioxidant defense capacity, thereby partially alleviating chondrocyte injury induced by inflammation and oxidative stress (47, 48). However, current evidence regarding ERα/ERβ mainly remains at the level of enhancing antioxidant capacity, maintaining mitochondrial function, and preserving ECM homeostasis in chondrocytes. Whether ERα/ERβ directly participate in the regulation of ferroptosis in chondrocytes remains to be further clarified.

#### 3.2.2 Estrogen-mediated regulation of ferroptosis-associated synovial inflammation in OA

Synovial inflammation is another core pathological feature of OA, and its occurrence is closely associated with the abnormal activation of fibroblast-like synoviocytes (FLSs) and immune cells such as macrophages (49, 50). In OA, synovial inflammation and ferroptosis may mutually reinforce each other, thereby forming a vicious cycle. On the one hand, the inflammatory microenvironment induces ferroptosis in chondrocytes and synovial cells by generating large amounts of reactive oxygen species (ROS) and pro-inflammatory cytokines, such as IL-1β and TNF-α, depleting key antioxidants such as glutathione (GSH), and suppressing the expression of GPX4 (51). On the other hand, within the arthritic synovial microenvironment, ferroptotic M2 macrophages can release the damage-associated molecular pattern molecule HMGB1. Extracellular HMGB1 further binds to TLR4 on the surface of M1 macrophages and activates STAT3 signaling, thereby amplifying inflammatory responses and exacerbating synovial inflammation and joint damage (52).

In this context, estrogen may indirectly modulate ferroptosis-associated synovial inflammation and the joint inflammatory microenvironment, primarily through its anti-inflammatory and antioxidant effects. *In vitro* studies have shown that estrogen can downregulate the expression of inflammatory mediators, including IL-1β and IL-6, in OA synovial cells by inhibiting NF-κB signaling (53). At the animal level, evidence also supports the regulatory role of estrogen in the synovial inflammatory microenvironment. In a mouse model of post-traumatic OA, ovariectomy aggravates joint pathological damage, whereas estrogen supplementation attenuates OA progression (54). In addition, estrogen receptor signaling has been shown to modulate inflammatory and autophagic networks in OA, for example, by affecting ER-related pathways to alleviate inflammatory responses, maintain cellular homeostasis, and inhibit excessive activation of inflammatory signaling such as the NLRP3 inflammasome (55). Although these changes do not directly prove that estrogen suppresses ferroptosis in synovial cells, they substantially overlap with the major upstream triggers of ferroptosis, including ROS accumulation, enhanced lipid peroxidation, mitochondrial stress, and collapse of the antioxidant defense system. Therefore, they support a plausible mechanistic connection between estrogen signaling and ferroptosis-related inflammatory injury in OA.

#### 3.2.3 Subchondral bone and the osteochondral unit: an intersection between OA and PMOP

OA is not merely a cartilage disease; abnormal subchondral bone remodeling also contributes importantly to OA pathogenesis. Subchondral bone sclerosis, altered trabecular architecture, bone marrow lesions, and vascular and neural abnormalities can change the mechanical environment and nutrient exchange within cartilage, thereby promoting cartilage degeneration. Postmenopausal estrogen deficiency can simultaneously affect cartilage metabolism and subchondral bone remodeling, suggesting that PMOP and OA intersect at the level of the “osteochondral unit”. Previous studies have shown that ovariectomy-induced estrogen deficiency aggravates cartilage degeneration and subchondral bone structural abnormalities in OA, whereas estrogen or estrogen receptor-related interventions can partially ameliorate osteochondral unit damage (56). In addition, subchondral osteoblast-derived factors such as SPARC have been reported to participate in bone–cartilage crosstalk in estrogen deficiency-related OA, suggesting that subchondral bone is an important regulatory component in postmenopausal OA progression (57).

However, a complete evidence chain linking “estrogen/estrogen receptors–ferroptosis–subchondral bone remodeling” is still lacking, as current direct evidence for the estrogen–ferroptosis axis in OA mainly comes from chondrocytes. Thus, in subchondral bone, estrogen deficiency may indirectly increase the susceptibility of the osteochondral unit to ferroptosis-related injury by disrupting bone–cartilage crosstalk, oxidative stress balance, and the inflammatory microenvironment, but whether it directly induces ferroptosis in subchondral bone cells requires further investigation.

### 3.3 The role of the estrogen–ferroptosis axis in IVDD

IVDD is an important pathological basis of chronic low back pain. Its development involves coordinated dysfunction among the nucleus pulposus (NP), annulus fibrosus (AF), cartilaginous endplate (CEP), and the local inflammatory and metabolic microenvironment. Compared with PMOP and OA, direct evidence for the “estrogen–ferroptosis axis” in IVDD remains relatively limited. However, the intervertebral disc is chronically exposed to an environment characterized by poor vascular supply, hypoxia, low nutrient availability, high lactate levels, and high mechanical loading, conditions that may collectively affect iron homeostasis, lipid peroxidation, and the GPX4-related antioxidant defense system (58, 59).

#### 3.3.1 Ferroptosis of nucleus pulposus cells in disc degeneration

Nucleus pulposus cells (NPCs) are key cells responsible for maintaining disc hydration and extracellular matrix (ECM) homeostasis. Studies have shown that oxidative stress can induce Fe²^+^ accumulation, increased lipid peroxidation, mitochondrial damage, and dysfunction of the SLC7A11/GPX4 antioxidant defense system in NPCs. Pharmacological interventions using ferroptosis inhibitors, such as ferrostatin-1 and deferoxamine, or ferroptosis inducers, such as RSL3, further demonstrate that ferroptosis contributes to NPC injury and IVDD progression (60, 61).

At the mechanistic level, impaired FPN-mediated iron export, disruption of the Se–SelK–GPX4 axis under mechanical overload, activation of the lactate–H3K18 lactylation–ACSL4 axis, and abnormalities in autophagy or chaperone-mediated autophagy can all aggravate NPC ferroptosis and ECM degradation by promoting iron retention, enhancing lipid peroxidation, or weakening the GPX4 system. These findings suggest that NPC ferroptosis is a network event jointly driven by iron homeostasis disruption, antioxidant system failure, metabolic reprogramming, and proteostasis impairment (62–64).

By contrast, direct evidence for the estrogen–ferroptosis axis in NPCs remains limited. Existing studies mainly show that ERα, ERβ, and GPER are expressed in intervertebral discs or NPCs, and that 17β-estradiol can promote NPC proliferation, inhibit apoptosis, alleviate oxidative injury, and maintain aggrecan and COL2A1 expression through ERβ–p38 MAPK, PI3K/Akt/FOXO3, PI3K/Akt/mTOR–p70S6K1, and autophagy-related pathways (5, 65–67). However, most of these studies did not systematically examine core ferroptosis indicators, such as Fe²^+^, lipid ROS, SLC7A11/GPX4, and ACSL4, nor did they provide reciprocal validation between estrogen/estrogen receptor interventions and ferroptosis modulators such as Fer-1, DFO, RSL3, or erastin. Therefore, estrogen signaling may increase the ferroptosis tolerance threshold of NPCs by maintaining mitochondrial function, reducing ROS accumulation, stabilizing the PI3K/Akt–mTOR–autophagy network, and protecting the GPX4-related antioxidant defense system. Strictly speaking, however, it is not yet possible to directly conclude that estrogen delays IVDD by inhibiting NPC ferroptosis.

#### 3.3.2 Cartilaginous endplate and annulus fibrosus cells

Existing studies have shown that cartilaginous endplate (CEP) cells can undergo iron homeostasis disruption and ferroptosis. Imbalance of the Nrf2/NCOA4 axis can enhance NCOA4-mediated ferritinophagy, leading to iron release, increased lipid peroxidation, mitochondrial dysfunction, extracellular matrix degradation, and endplate degeneration or calcification (68). In addition, recent evidence indicates that oxidative stress can activate the YAP/TEAD1/NCOA4 axis, thereby promoting NCOA4-dependent ferritinophagy and ferroptosis in endplate chondrocytes and aggravating intervertebral disc degeneration, further supporting ferroptosis as an active mechanism in CEP degeneration rather than a merely secondary phenomenon (69). Meanwhile, estrogen has been reported to exert protective effects on the cartilaginous endplate. 17β-estradiol can promote CEP cell proliferation, inhibit apoptosis and inflammatory factor expression, and maintain Aggrecan and COL2A1 levels, partly through ERα-related mechanisms and miR-221 regulation (70). Estrogen deficiency can aggravate endplate calcification and intervertebral disc degeneration, whereas estrogen supplementation may partially alleviate these changes (71). However, these studies have not directly demonstrated that estrogen protects the cartilaginous endplate by inhibiting ferroptosis. Thus, at present, estrogen signaling and CEP ferroptosis can only be considered to have mechanistic intersections at the levels of ROS regulation, Nrf2-mediated defense, inflammation, iron metabolism, ferritinophagy, and calcification.

In the annulus fibrosus (AF), evidence for ferroptosis mainly comes from oxidative stress models. TBHP can induce iron accumulation, lipid peroxidation, and mitochondrial damage in AF cells, while ferrostatin-1 or deferoxamine can alleviate these changes, suggesting that AF degeneration is not driven solely by mechanical rupture but is also accompanied by oxidative lipid injury and iron metabolism remodeling (60). Some transcriptomic studies further suggest that antioxidant- or ferroptosis-related genes, such as MGST1 and CREB3, may participate in AF degeneration (72, 73). Evidence also indicates that estrogen signaling is biologically relevant in the annulus fibrosus. ERβ is expressed in human annulus cells, and 17β-estradiol has been reported to promote annulus cell proliferation (74). In rat annulus fibrosus cells (AFCs), 17β-estradiol inhibits IL-1β-induced apoptosis in a dose- and time-dependent manner, and its protective effect involves estrogen receptor signaling and α1 integrin-mediated adhesion to type I collagen (75, 76). Nevertheless, direct AF-specific evidence linking estrogen signaling to ferroptosis remains limited.

From the perspective of the overall microenvironment, mechanical overload, lactate accumulation, hyperhomocysteinemia, and chronic inflammation can all increase the ferroptosis susceptibility of disc cells by promoting ROS accumulation, weakening the SLC7A11/GPX4 defense system, or enhancing ACSL4-dependent lipid peroxidation (63, 64). Therefore, estrogen deficiency may indirectly promote a ferroptosis-susceptible state in CEP, AF, and nucleus pulposus cells by weakening the endplate barrier and amplifying inflammatory-oxidative stress and metabolic pressure. However, whether the “estrogen–ferroptosis axis” directly drives pathological processes in the cartilaginous endplate and annulus fibrosus remains to be further investigated.

## 4 Comorbid links and mechanistic intersections among PMOP, OA, and IVDD

Although osteoporosis, osteoarthritis, and intervertebral disc degeneration are traditionally classified as a bone loss disease, a degenerative joint disease, and a degenerative spinal disorder, respectively, they often show substantial clinical overlap in postmenopausal women and older adults. These conditions commonly manifest as degeneration of bone–cartilage–intervertebral disc structures, chronic pain, reduced motor function, and impaired quality of life. Therefore, they may be better understood as heterogeneous responses of different tissues to a shared background of endocrine disturbance, inflammation, oxidative stress, and mechanical abnormalities (2) (**Table 2**).

**Table 2:** Shared mechanisms and tissue-specific differences of the estrogen–ferroptosis axis in PMOP, OA, and IVDD.

Mechanistic dimension	Shared mechanisms among the three diseases	PMOP-specific features	OA-specific features	IVDD-specific features
Estrogen deficiency and cellular vulnerability	Estrogen decline weakens cellular resistance to oxidative stress, iron dyshomeostasis, and ferroptosis-related injury	Estrogen deficiency directly disrupts bone remodeling and reduces the anti-ferroptotic capacity of osteogenic lineage cells	Estrogen deficiency promotes cartilage degeneration and inflammatory activation, contributing to postmenopausal OA progression	Estrogen deficiency may reduce the stress resistance of disc cells, thereby accelerating degeneration under adverse microenvironmental conditions
Iron dyshomeostasis	Iron accumulation or impaired iron handling increases ferroptosis susceptibility across skeletal and joint tissues	Iron imbalance mainly affects osteoblasts, BMSCs, and osteocyte-mediated osteoclast activation and bone remodeling processes	Fe²^+^ accumulation in chondrocytes contributes to lipid ROS production and cartilage matrix degradation	Iron accumulation in nucleus pulposus and endplate-related cells is associated with mitochondrial damage and degenerative changes
Lipid peroxidation and ROS accumulation	Increased lipid ROS and oxidative stress represent central execution events of ferroptosis	Lipid peroxidation impairs osteogenic differentiation and contributes to insufficient bone formation	Lipid ROS promotes chondrocyte injury, ECM degradation, and upregulation of catabolic enzymes	Lipid peroxidation occurs in a low-nutrient, hypoxic, and mechanically loaded disc microenvironment, amplifying cell stress
SLC7A11/GPX4 antioxidant defense	Suppression of the SLC7A11/GPX4 axis reduces ferroptosis resistance in all three disorders	SLC7A11/GPX4 downregulation is closely linked to impaired osteoblast survival and osteogenic function	SLC7A11/GPX4 inhibition is associated with chondrocyte ferroptosis and ECM loss	SLC7A11/GPX4 downregulation, together with ACSL4 upregulation, may contribute to ferroptosis-prone disc degeneration
Inflammation–ferroptosis interaction	Inflammatory signaling and ferroptosis may reinforce each other through ROS generation, matrix catabolism, and tissue damage	Inflammation may aggravate bone microenvironmental imbalance and affect bone remodeling	Synovial inflammation and chondrocyte ferroptosis may jointly amplify cartilage destruction and pain-related pathology	Inflammatory stress may interact with nutrient deprivation and mechanical loading to worsen disc cell injury
Matrix and tissue structural damage	Ferroptosis-related cell injury contributes to extracellular matrix or skeletal tissue deterioration	Loss of osteogenic cell function contributes to reduced bone formation, trabecular deterioration, and bone fragility	Chondrocyte ferroptosis reduces ECM synthesis and promotes cartilage thinning and matrix degradation	Ferroptosis-prone disc cell injury contributes to nucleus pulposus matrix loss, endplate calcification, and annulus fibrosus disruption

### 4.1 Shared tissue vulnerabilities underlying the co-occurrence of PMOP, OA, and IVDD

The comorbidity among PMOP, OA, and IVDD may be attributed to shared histological vulnerabilities of bone, articular cartilage, and intervertebral disc tissues. These tissues are all located in load-bearing systems and are chronically exposed to mechanical stress. Physiological mechanical loading is essential for maintaining tissue homeostasis and adaptive remodeling, whereas excessive, repetitive, or abnormal loading may exceed the adaptive capacity of these tissues and become a source of injury. Experimental studies have shown that fatigue loading induces microdamage and osteocyte apoptosis in bone (77), while excessive mechanical stress promotes chondrocyte apoptosis and cartilage degeneration (78). Abnormal mechanical loading associated with spinal instability can likewise accelerate intervertebral disc degeneration (71, 79).

Importantly, mechanical stimulation and estrogen signaling are not independent factors but are associated with each other. In bone cells, both mechanical strain and estrogen can activate ERα (80), and osteocytes use ERα to respond to mechanical strain, while cellular ERα levels are themselves regulated by estrogen (81). These findings indicate that estrogen signaling and mechanotransduction converge at the level of ERα and suggest that estrogen deficiency may alter the adaptive responsiveness of bone cells to mechanical loading. A similar interaction has been observed in articular cartilage, where GPER attenuates mechanical stress-induced chondrocyte apoptosis by suppressing Piezo1-mediated mechanotransduction (82). Moreover, in the intervertebral disc, estrogen deficiency aggravates spinal instability-induced degeneration, whereas 17β-estradiol supplementation attenuates these changes (71). Collectively, these findings suggest that estrogen deficiency may act as a susceptibility amplifier under abnormal mechanical loading, increasing the vulnerability of musculoskeletal tissues to mechanically induced injury.

Meanwhile, bone, articular cartilage, and intervertebral disc tissues share a strong dependence on extracellular matrix homeostasis. Impaired bone matrix formation and mineralization contribute to PMOP; cartilage matrix degradation promotes OA progression and is closely associated with subchondral bone remodeling; and proteoglycan loss, collagen disorganization, and cartilaginous endplate abnormalities reduce disc hydration and compressive resistance in IVDD (83–85). In addition, these tissues generally have limited intrinsic repair capacity. Articular cartilage is poorly vascularized, the intervertebral disc is nutrient-poor and cell-poor, and bone remodeling depends on coordinated cellular activity within the bone marrow microenvironment (86, 87). Together, these shared tissue vulnerabilities may render bone, articular cartilage, and intervertebral disc more susceptible to estrogen deficiency-induced disruption of tissue homeostasis during menopause, thereby facilitating the co-occurrence of PMOP, OA, and IVDD.

### 4.2 Estrogen deficiency: a shared upstream factor in postmenopausal comorbidity

The decline in estrogen levels after menopause is a key factor in the development of PMOP and may also increase tissue vulnerability to OA and IVDD. In bone tissue, estrogen deficiency not only promotes increased bone resorption and reduced bone formation, but also alters the bone marrow microenvironment, shifting it toward a state of inflammation and oxidative stress (88). In joints and intervertebral discs, the effect of estrogen deficiency is more characterized by “susceptibility amplification”. In OA, the increased risk of developing OA, greater symptom burden, and changes in articular cartilage and subchondral bone observed in postmenopausal women suggest that estrogen signaling plays a protective role in joint homeostasis (89). In IVDD, early epidemiological and imaging studies have found that menopause is associated with more severe lumbar disc degeneration, suggesting that relative estrogen deficiency may be an important background factor accelerating intervertebral disc degeneration in postmenopausal women (90). Animal studies have also shown that estrogen deficiency aggravates spinal instability-induced intervertebral disc degeneration, whereas estrogen supplementation exerts a certain protective effect (71).

Overall, estrogen deficiency may represent a common factor contributing to the development and progression of PMOP, OA, and IVDD. Beyond the reduction in circulating estrogen levels, estrogen deficiency impairs estrogen-mediated protective responses in multiple musculoskeletal tissues, leading to increased oxidative stress, inflammation, ferroptosis susceptibility, and extracellular matrix disruption. These shared molecular alterations may help explain the increased susceptibility to, and coexistence of, these degenerative disorders after menopause.

### 4.3 Ferroptosis: a shared mode of cellular injury in bone–joint–intervertebral disc degeneration

From the perspective of ferroptosis, estrogen deficiency may increase the susceptibility of different musculoskeletal cell types by weakening anti-ferroptotic defenses through cell-specific pathways that ultimately converge on common molecular processes (91). In PMOP, ferroptosis predominantly impairs osteoblast and BMSC survival and osteogenic capacity, thereby reducing bone formation (92–94). In OA, ferroptosis disrupts chondrocyte homeostasis and promotes extracellular matrix degradation (95–98). In IVDD, ferroptosis of nucleus pulposus, annulus fibrosus, and cartilaginous endplate cells contributes to cellular dysfunction and matrix degeneration under oxidative, metabolic, and mechanical stress (60, 61). Despite these cell-specific differences, these effects converge on several core processes of ferroptosis, particularly impaired antioxidant defense, disturbed intracellular iron homeostasis, and increased susceptibility to membrane lipid peroxidation. Thus, estrogen deficiency may enhance ferroptosis susceptibility through different upstream pathways in different cell types, while ultimately converging on a shared ferroptotic machinery.

### 4.4 Inflammation–ferroptosis crosstalk: an amplification loop in comorbidity progression

Aging and metabolic disturbances are commonly associated with chronic low-grade inflammation, which provides a shared inflammatory background for PMOP, OA, and IVDD. In the postmenopausal setting, estrogen deficiency may further amplify this inflammatory state by weakening estrogen-mediated anti-inflammatory regulation. For example, 17β-estradiol has been shown to inhibit IL-6 production and NF-κB transcriptional activity in human monocytes through estrogen receptor-mediated signaling (99). Conversely, estrogen deficiency increases the production of TNF-α by T cells, while ovariectomy promotes the expansion of TNF-α-producing T cells and Th17 cells and increases TNF-α and IL-17 signaling in the bone marrow (100, 101). These findings suggest that estrogen deficiency may reinforce chronic inflammation by weakening estrogen-mediated suppression of inflammatory signaling and enhancing pro-inflammatory immune-cell activity. Under this chronic inflammatory state, inflammatory mediators and catabolic factors, including IL-1β, IL-6, TNF-α, PGE2, COX-2, MMPs, and ADAMTS, can remain elevated in the local microenvironments of bone marrow, joints, and intervertebral discs (102, 103). On the one hand, these inflammatory signals promote osteoclast activation, cartilage matrix degradation, and intervertebral disc ECM destruction through pathways such as NF-κB, MAPK, JAK/STAT, and the NLRP3 inflammasome (104). On the other hand, these inflammatory changes can exacerbate ferroptosis by increasing ROS generation, disrupting iron homeostasis, depleting GSH, and suppressing the SLC7A11/GPX4-mediated anti-lipid peroxidation defense system (105).

More importantly, ferroptosis is not merely a downstream consequence of inflammation. Once ferroptotic cells undergo membrane lipid peroxidation and loss of membrane integrity, they can release 4-HNE, MDA, oxidized phospholipids, HMGB1, and other DAMPs. These signals can be recognized by macrophages, synovial cells, bone marrow immune cells, and local immune cells within the intervertebral disc, further inducing the production of inflammatory cytokines such as IL-1β, IL-6, and TNF-α. This process forms a vicious cycle of “inflammation-induced ferroptosis–ferroptosis-driven inflammatory amplification” (106, 107). Therefore, inflammation can create permissive conditions for ferroptosis by enhancing oxidative stress, increasing iron burden, and weakening anti-lipid peroxidation defenses; conversely, ferroptosis can reactivate inflammatory responses through the release of lipid peroxidation products and DAMPs, driving local injury from a transient stress response toward a persistent degenerative process (105).

**Figure 3:**
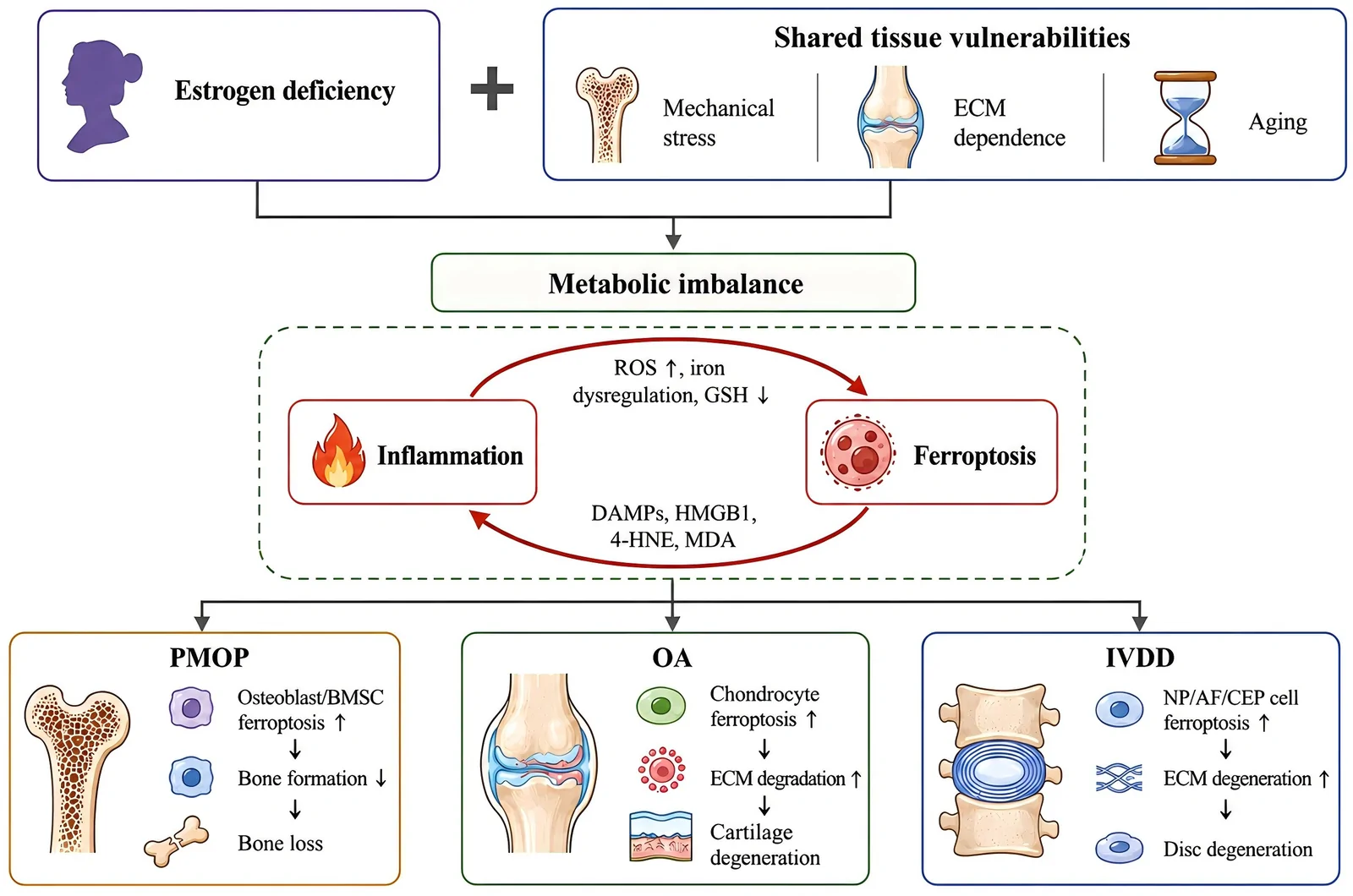
Shared estrogen deficiency–ferroptosis network linking PMOP, OA, and IVDD. Estrogen deficiency and shared tissue vulnerabilities may contribute to metabolic imbalance and enhance the interplay between inflammation and ferroptosis, thereby increasing susceptibility to cell-specific ferroptotic injury and tissue degeneration in PMOP, OA, and IVDD.

This loop is supported by varying degrees of evidence in PMOP, OA, and IVDD. In PMOP, chronic inflammation and oxidative stress can disrupt bone remodeling balance, while osteoblast ferroptosis, impairment of the GPX4 defense system, and lipid peroxidation accumulation may further disturb the bone marrow microenvironment (30). In OA, evidence for inflammation–ferroptosis crosstalk is more direct. IL-1β and iron burden can induce ROS accumulation and ferroptosis-related molecular changes in chondrocytes, whereas DAMPs generated by ferroptotic chondrocytes can further activate synovial cells and local immune responses, promoting the sustained release of inflammatory mediators and ROS, thereby amplifying synovitis, chondrocyte injury, and ECM degradation (108). A similar feedback loop also exists in IVDD. Inflammatory factors and oxidative stress can induce ROS accumulation, iron homeostasis disruption, and downregulation of the SLC7A11/GPX4 defense system in nucleus pulposus cells and annulus fibrosus cells, thereby promoting lipid peroxidation and ferroptosis (60). Oxidized lipids and DAMPs released by ferroptotic cells can in turn activate local inflammatory responses within the intervertebral disc and maintain the sustained expression of IL-1β, TNF-α, MMPs, and other mediators. Recent studies have further conceptualized IVDD as a vicious cycle mutually driven by ROS, ferroptosis, and inflammation, and have shown that simultaneous ROS scavenging, ferroptosis inhibition, and inflammation alleviation may contribute to intervertebral disc repair (109). Therefore, in the comorbid progression of PMOP, OA, and IVDD, inflammation is not simply a consequence of tissue injury, but a key factor that increases ferroptosis susceptibility and is reciprocally amplified by ferroptosis. Inflammation–ferroptosis crosstalk may shift local injury in bone, articular cartilage, and intervertebral discs from transient stress to persistent degeneration, thereby promoting comorbidity progression (**Figure 3**).

## 5 Therapeutic strategies and translational prospects targeting the estrogen–ferroptosis axis

Given the shared pathological features of PMOP, OA, and IVDD, including oxidative stress, inflammatory activation, extracellular matrix dysregulation, and impaired tissue remodeling, the estrogen–ferroptosis axis may represent an important mechanistic intersection in postmenopausal multi-tissue degeneration. Therefore, therapeutic strategies targeting this axis may offer a promising approach for coordinated intervention across PMOP, OA, and IVDD (**Table 3**).

**Table 3:** Therapeutic strategies, potential targets, applicable scenarios, and translational limitations for targeting the estrogen–ferroptosis axis.

Therapeutic strategy	Main targets/mechanisms	Potential applicable scenarios	Major translational limitations
Estrogen replacement therapy / MHT	Restores estrogen signaling and may enhance antioxidant defense and GPX4-related ferroptosis resistance	Prevention or management of postmenopausal bone loss in appropriately selected women; potential modulation of tissue vulnerability in OA and IVDD	Systemic hormone-related risks; insufficient direct anti-ferroptosis evidence in OA and IVDD
SERMs, TSECs, and receptor subtype targeting	Selectively modulates ERα, ERβ, or GPER/GPR30 while reducing systemic estrogen exposure	Established bone-protective use of selected SERMs in PMOP; experimental receptor-subtype targeting in OA and IVDD	Receptor subtype functions are tissue-dependent; anti-ferroptotic mechanisms require further validation
Anti-iron accumulation / iron homeostasis regulation	Limits labile Fe²^+^ accumulation and targets iron-handling pathways involving FPN, FTH1, TfR, and ferritinophagy	PMOP, OA, and IVDD associated with iron overload or iron dyshomeostasis	Excessive intervention may disturb physiological iron metabolism and hematopoiesis
Enhancement of antioxidant defense	Activates antioxidant defense axes such as Nrf2/GPX4 and SLC7A11–GSH–GPX4	Oxidative stress-related injury in bone, cartilage, and intervertebral disc cells	Long-term or non-selective antioxidant activation may interfere with physiological ROS signaling
Inhibition of lipid peroxidation	Limits lipid peroxide accumulation, preserves GPX4-dependent defense, and/or suppresses ACSL4-associated lipid peroxidation	Osteogenic cell injury in PMOP, cartilage degeneration in OA, and lipid oxidative injury in IVDD	Most agents remain at the preclinical stage; limited tissue specificity
Local delivery, nanomaterials, and biomaterial-based strategies	Increases local drug concentrations through hydrogels, nanoparticles, or targeted carriers	Intra-articular delivery for OA, intradiscal delivery for IVDD, and systemic bone-targeted delivery for PMOP	Material safety, release stability, and clinical standardization remain unresolved
Exosome- and nucleic acid-based delivery strategies	Uses exosomes, miRNAs, siRNAs, or other nucleic-acid cargos to modulate ferroptosis-related pathway	NPC protection in IVDD and cartilage-directed therapy in OA; potential future application to bone-targeted therapy in PMOP	Targeting efficiency, cargo stability, and quality control remain major barriers

### 5.1 Estrogen-based and estrogen receptor–targeted strategies

Restoration or selective modulation of estrogen signaling represents the most upstream therapeutic approach within the estrogen–ferroptosis axis. Menopausal hormone therapy (MHT) is the most direct strategy and has an established clinical role in preventing postmenopausal bone loss and reducing fracture risk. According to the 2022 position statement of the North American Menopause Society, the benefit–risk profile of MHT is generally favorable for women younger than 60 years or within 10 years of menopause onset in the absence of contraindications, although treatment should be individualized according to formulation, dose, route, duration, and progestogen use (110). Mechanistically, emerging evidence that estrogen regulates GPX4 expression in osteoblasts suggests a potential link between its endocrine effects and ferroptosis resistance (30). In OA, although postmenopausal estrogen decline is associated with increased disease risk, there is no consistent evidence that MHT exerts a definitive disease-modifying effect (111). Current studies more strongly support a role for estrogen signaling in regulating cartilage homeostasis, inflammation, and matrix metabolism. In IVDD, estrogen deficiency has been shown to aggravate disc degeneration, while 17β-estradiol supplementation may alleviate degeneration through antioxidant effects, autophagy regulation, and pathways such as ERβ/NF-κB (112, 113). Nevertheless, these studies have not systematically evaluated ferroptosis-related markers. Therefore, estrogen-based therapy in OA and IVDD is currently better defined as a potential modulator of susceptibility or microenvironmental regulation, rather than a well-established anti-ferroptosis treatment.

Due to the potential risks associated with systemic estrogen therapy, selective estrogen receptor modulators (SERMs) and tissue-selective estrogen complexes (TSECs) offer a safer translational approach for targeting the “estrogen–ferroptosis axis”. Among these, SERMs have the most well-established clinical foundation in PMOP. For example, raloxifene can inhibit osteoclastogenesis, improve bone turnover, and reduce the risk of vertebral fractures through ER-related signaling pathways (114, 115). In OA, receptor-subtype targeting may be particularly relevant, as reduced GPER expression has been observed in OA cartilage and GPER activation can suppress chondrocyte ferroptosis and ameliorate cartilage degeneration (16). In IVDD, 17β-estradiol has shown protective effects through ERβ-related signaling, while raloxifene has been reported to ameliorate structural, biomechanical, and degeneration-related changes in experimental models (113, 116, 117). Nevertheless, with the exception of emerging evidence for GPER-mediated ferroptosis regulation in OA, direct evidence that SERMs or estrogen receptor subtype–selective interventions regulate canonical ferroptosis nodes such as GPX4, SLC7A11, FTH1, FPN, or Nrf2 remains insufficient. Overall, the potential value of SERMs, TSECs, and ERα/ERβ/GPER subtype targeting lies in their ability to modulate estrogen signaling in a tissue-selective manner, potentially limit systemic estrogen-related adverse effects, and provide a framework for developing more targeted interventions across PMOP, OA, and IVDD.

### 5.2 Therapeutic strategies targeting the execution phase of ferroptosis

Within the framework of the “estrogen–ferroptosis axis”, direct inhibition of ferroptosis should be regarded as a shared downstream intervention strategy for PMOP, OA, and IVDD. Existing studies have provided preliminary support for this approach across the three conditions. First, targeting iron overload and disrupted iron homeostasis can exert protective effects in bone, cartilage, and intervertebral disc degeneration. In osteoporosis, iron overload promotes lipid peroxidation in osteoblasts and bone loss through pathways such as IRP1/NOX4, while interventions with ferrostatin-1 or deferoxamine can attenuate these effects, supporting the bone-protective value of iron regulation (10, 118). In OA, ferrostatin-1 and deferoxamine have been shown to inhibit disease progression both *in vitro* and *in vivo*, with GPX4 identified as a key regulator of chondrocyte ferroptosis and ECM homeostasis (119). In IVDD, iron overload induces oxidative stress and ferroptosis in cartilaginous endplate cells, thereby promoting disc degeneration and endplate calcification. Interventions with deferoxamine, N-acetylcysteine, or ferrostatin-1 can mitigate iron-induced IVDD changes (120), while ferrostatin-1 and deferoxamine also suppress oxidative stress-induced ferroptosis in annulus fibrosus and nucleus pulposus cells (60).

Second, targeting lipid peroxidation represents a shared intervention strategy at the execution phase across these diseases. In PMOP, estrogen deficiency reduces GPX4 expression in osteoblasts and enhances phospholipid peroxidation; clearance of lipid peroxides or activation of GPX4 can improve osteogenic impairment and bone loss (30). In OA, ferrostatin-1 alleviates chondrocyte death and ECM degradation by limiting lipid peroxidation (119). In IVDD, oxidative stress induces increased lipid peroxidation and ferroptosis in annulus fibrosus and nucleus pulposus cells, whereas interventions such as ferrostatin-1, deferoxamine, or polydopamine nanoparticles can reduce lipid peroxidation damage and attenuate disc degeneration (60, 121).

Third, targeting the SLC7A11/GSH/GPX4 defense axis represents a core cross-disease strategy to restore anti-ferroptosis capacity. In osteoporosis, activation of VDR or the SLC7A11/GPX4 pathway can reduce osteoblast ferroptosis and improve bone loss (10). In OA, GPX4 downregulation increases chondrocyte susceptibility to oxidative stress and exacerbates ECM degradation, whereas interventions such as Fer-1, DFO, or modulation of SLC7A11/GPX4 signaling exert protective effects on cartilage (119, 122). In IVDD, the SLC7A11/GPX4 axis is involved in nucleus pulposus cell ferroptosis and ECM metabolic imbalance. Inhibition of SLC7A11 promotes ferroptosis in these cells, while maintaining GPX4 levels and reducing Fe²^+^ and MDA can alleviate nucleus pulposus cell injury and disc degeneration (121).

In summary, although PMOP, OA, and IVDD occur in different tissues, current evidence suggests that all three-share key ferroptosis-related features, including iron homeostasis imbalance, enhanced lipid peroxidation, and insufficient SLC7A11/GSH/GPX4-mediated defense. This indicates that ferroptosis may represent a common downstream execution program underlying postmenopausal degeneration of bone, cartilage, and intervertebral discs. However, significant differences exist among these tissues in terms of iron metabolism activity, membrane lipid composition, and regenerative capacity. Future studies should therefore evaluate ferroptosis-related markers in unified cross-disease models to determine whether anti-ferroptosis interventions can evolve from single-disease protective effects into a shared therapeutic strategy for PMOP, OA, and IVDD.

### 5.3 Targeted delivery, biomaterials, and exosome-based strategies

Although estrogen modulation and anti-ferroptosis agents hold therapeutic potential, the affected tissues in PMOP, OA, and IVDD differ substantially in vascular supply, extracellular matrix composition, mechanical environment, and drug accessibility. Therefore, targeted delivery should be tailored to the anatomical and biological characteristics of each tissue. Tissue-specific accumulation may be achieved through several complementary mechanisms, including local administration to anatomically restrict drug distribution, active targeting through ligand–matrix or ligand–receptor interactions, physicochemical interactions that enhance tissue penetration or cellular uptake, and biomaterial-mediated retention and sustained release. These approaches may increase local exposure to anti-ferroptosis agents while reducing systemic estrogen exposure and interference with physiological iron metabolism.

In OA, articular cartilage and chondrocytes represent the principal targets. Intra-articular injection provides direct anatomical localization within the joint, but rapid clearance from the synovial cavity and the dense avascular cartilage matrix remain major barriers. Active cartilage targeting can be achieved by surface modification of nanocarriers with cartilage-binding ligands. For example, the WYRGRL peptide specifically binds type II collagen in the cartilage matrix, and WYRGRL-functionalized nanoparticles show markedly enhanced cartilage localization and retention following intra-articular administration (123). Such ligand-based platforms could potentially be combined with ferroptosis-targeted cargos. More directly, a 2025 study developed chitosan-modified starlike Au nanoparticles for intra-articular delivery of siDDIT3, in which the positively charged surface enhanced chondrocyte uptake and siRNA transfection, while photothermal activation further improved the local therapeutic effect. This system suppressed DDIT3-associated ferroptosis and attenuated cartilage degeneration in OA models (124). Thus, cartilage-directed delivery can combine anatomical localization, matrix-specific ligand recognition, and enhanced cellular uptake to improve the local efficacy of anti-ferroptosis interventions (125).

In IVDD, NPCs represent an important cellular target, while the avascular nature of the intervertebral disc limits effective systemic drug delivery. Therefore, intradiscal administration combined with local retention systems may be particularly suitable. For example, an injectable ECM-mimetic HACS hydrogel was used to deliver miR-221-3p-enriched, CPP-modified nucleus pulposus progenitor cell-derived exosomes directly into the nucleus pulposus cavity (126). The hydrogel prolonged local exosome retention and sustained release, while CPP modification enhanced cellular uptake by NPCs. This combined delivery strategy suppressed NPC ferroptosis through the miR-221-3p/IRF8/STAT1 axis and promoted disc repair (126). Polydopamine nanoparticles provide another anti-ferroptosis approach by scavenging ROS, chelating iron ions, and preventing GPX4 ubiquitination and degradation (121), although further ligand engineering may be required to improve cell specificity.

For PMOP, systemic administration remains more feasible because bone is well vascularized; however, efficient accumulation in bone remains critical for minimizing off-target exposure. Bone-targeting carriers can exploit the high affinity of bisphosphonates such as alendronate for hydroxyapatite, the major mineral component of bone. For example, alendronate-modified PLGA nanoparticles exhibit enhanced hydroxyapatite binding and preferential delivery to the bone surface, demonstrating a practical mechanism for bone-specific accumulation (127). More importantly, a recent study developed alendronate-conjugated selenium-doped carbon dots (ASCDs) specifically for PMOP (128). These nanoparticles combined alendronate-mediated bone targeting with selenium-based anti-ferroptosis activity. ASCDs suppressed ferroptosis in osteoblasts and BMSCs by activating the system Xc^−^/GSH/GPX4 antioxidant pathway and showed greater efficacy than non-targeted selenium-doped carbon dots in ovariectomized mice, including restoration of GPX4 expression, reduction of bone ferroptosis, and improvement of trabecular bone loss (128). This provides direct evidence that bone-homing ligands can be integrated with ferroptosis-modulating nanocarriers for targeted intervention in PMOP.

Overall, targeted delivery across PMOP, OA, and IVDD should be designed according to tissue-specific barriers rather than relying on a single delivery principle. For OA and IVDD, local intra-articular or intradiscal administration can provide anatomical targeting, which may be further strengthened by cartilage-binding ligands, cell-penetrating peptides, or hydrogel-mediated retention. For PMOP, systemic bone-homing strategies based on hydroxyapatite-binding ligands may facilitate preferential skeletal accumulation. Future studies could further integrate these delivery principles with estrogen receptor subtype modulators, Nrf2/GPX4 activators, iron chelators, lipid peroxidation inhibitors, or nucleic acid therapies to achieve more selective regulation of the estrogen–ferroptosis axis while minimizing systemic adverse effects.

### 5.4 Challenges in clinical translation and future directions

Although targeting the estrogen–ferroptosis axis provides a new conceptual framework for the coordinated treatment of PMOP, OA, and IVDD, the field is still in an early stage of transitioning from mechanistic insights to translational application. First, current evidence shows clear imbalance across diseases and tissues. In PMOP, the mechanistic link between estrogen deficiency, ferroptosis in osteoblasts/BMSCs, and bone loss is relatively well supported (30, 35, 36). In OA, evidence linking chondrocyte ferroptosis to ECM degradation is rapidly accumulating (119). However, in IVDD, direct evidence connecting “estrogen/estrogen receptors–ferroptosis–disc degeneration” remains limited (121). Notably, many studies in OA and IVDD focus on the anti-inflammatory, antioxidant, anti-apoptotic, or ECM-stabilizing effects of estrogen, without systematically assessing key ferroptosis markers such as Fe²^+^, MDA, 4-HNE, C11-BODIPY, GPX4, SLC7A11, ACSL4, FTH1, FPN, and mitochondrial morphology (5, 46, 67).

Second, the estrogen–ferroptosis axis exhibits strong cell type– and disease stage–specific characteristics. In PMOP, inhibiting ferroptosis in osteoblasts and BMSCs is generally protective, whereas in osteoclasts, ferroptosis may exert stage-dependent biphasic effects. In OA, protecting chondrocytes from ferroptosis helps maintain ECM homeostasis, but ferroptosis in synovial macrophages or FLS may have more complex relationships with inflammation. In IVDD, nucleus pulposus, annulus fibrosus, and cartilaginous endplate cells exist in distinct nutritional, mechanical, and redox environments, and may differ in ferroptosis thresholds and responsiveness to estrogen signaling. Therefore, future therapies should not aim for global inhibition of ferroptosis, but instead tailor interventions based on cell type, disease stage, and local microenvironment.

Third, patient stratification and biomarker systems are critical for clinical translation (129). Not all patients with PMOP, OA, or IVDD exhibit the same degree of estrogen deficiency, iron dysregulation, or lipid peroxidation. Future efforts should focus on identifying populations most likely to benefit from targeting this axis, such as early postmenopausal women, patients with PMOP combined with OA or IVDD, individuals with elevated iron load or oxidative stress, and those with imaging evidence of multi-site degeneration across bone, cartilage, and intervertebral discs. Potential stratification markers may include estradiol, ferritin, transferrin saturation, hepcidin, bone turnover markers, inflammatory cytokines, oxidative lipid metabolites, and ferroptosis-related indicators such as 4-HNE, MDA, GPX4, SLC7A11, ACSL4, FTH1, and FPN in tissues or body fluids.

Finally, safety and clinical endpoint design remain key considerations. Long-term use of estrogen or estrogen-like agents carries risks related to breast and endometrial tissues, thrombosis, and cardiovascular events (130). Iron chelators may interfere with normal iron metabolism and hematopoiesis (131), while potent antioxidant or GPX4-activating strategies may disrupt physiological ROS signaling and tissue repair processes (132). Therefore, the optimal strategy is not prolonged, systemic, and non-selective inhibition of ferroptosis, but rather the development of approaches characterized by receptor subtype selectivity, local delivery, short-term intervention, and biodegradable intelligent materials. Future studies integrating cross-disease models in postmenopausal settings, combined with validation in clinical samples, will be essential to advance this field from mechanistic association toward true translational application.

## 6 Discussion

This review proposes the estrogen–ferroptosis axis as a potential integrative framework to interpret postmenopausal musculoskeletal degeneration across osteoporosis, osteoarthritis, and intervertebral disc degeneration. Rather than a single linear pathway, this axis integrates endocrine decline, iron dysregulation, lipid peroxidation, and redox imbalance into a network of tissue vulnerability. In this context, estrogen deficiency may lower the threshold for ferroptosis-related injury in multiple musculoskeletal cell types, thereby contributing to multi-tissue degeneration under shared systemic conditions (133, 134).

Importantly, current evidence is characterized by a gradient of mechanistic maturity across diseases rather than simple inconsistency. In postmenopausal osteoporosis, the link between estrogen deficiency, impaired GPX4 signaling, lipid peroxidation, and osteoblast/BMSC dysfunction is relatively well supported (30, 135). In osteoarthritis, increasing evidence—particularly involving GPER signaling—suggests a direct role of estrogen in modulating chondrocyte ferroptosis, although data in synovium and subchondral bone remain limited (16). In intervertebral disc degeneration, ferroptosis has been identified in nucleus pulposus and endplate cells, but direct experimental evidence connecting estrogen signaling to ferroptosis regulation is still scarce (136). Thus, this axis is currently best regarded as well supported in bone, expanding in cartilage, and at an early exploratory stage in disc tissues.

These differences highlight the context-dependent nature of ferroptosis. Its role varies across cell types, tissues, and disease stages, suggesting that ferroptosis may require precise, context-specific modulation rather than uniform inhibition (137). Instead, future studies need to define when and where ferroptosis modulation is beneficial under specific microenvironmental conditions, which may ultimately enable more targeted and stage-adapted therapeutic strategies.

From a translational perspective, targeting this axis presents both opportunities and limitations. Estrogen-based therapies may restore upstream regulation but are constrained by risks such as hormone-sensitive cancers and thromboembolic events (138). While these limitations restrict systemic application, they also underscore the need for tissue-selective estrogen signaling modulators or downstream pathway-targeted approaches. Ferroptosis inhibitors and iron-modulating strategies show promise in preclinical models, yet long-term systemic inhibition may disrupt physiological iron homeostasis, immune regulation, and tumor surveillance (139). This further highlights the importance of developing localized, biomaterial-assisted, or cell-specific delivery strategies, rather than generalized systemic approaches. Future research should focus on integrated postmenopausal models evaluating bone, cartilage, and disc simultaneously, together with standardized ferroptosis markers (e.g., Fe²^+^, lipid ROS, GPX4, SLC7A11) and receptor-specific studies to clarify the roles of ERα, ERβ, and GPER in ferroptosis regulation. In addition, biomarker-guided patient stratification will be essential for clinical translation.

Overall, the estrogen–ferroptosis axis should be considered a hypothesis-generating and integrative framework, rather than a fully established universal mechanism. Despite current limitations, it provides a promising conceptual basis for understanding how endocrine decline and metabolic stress converge on regulated cell injury, and may open new avenues for cross-tissue and precision therapeutic strategies in postmenopausal musculoskeletal disorders.

## 7 Conclusion

In summary, this review proposes the estrogen–ferroptosis axis as a potential integrative framework to interpret the shared yet tissue-specific degeneration observed in PMOP, OA, and IVDD. Rather than acting as an isolated pathogenic pathway, this axis integrates endocrine decline, iron dysregulation, lipid peroxidation, and inflammatory amplification into a coordinated network that drives multi-tissue vulnerability under postmenopausal conditions.

This perspective shifts the understanding of these disorders from independent diseases toward interconnected manifestations of a common systemic imbalance, and highlights the potential of multi-level, cross-tissue therapeutic strategies targeting ferroptosis-related pathways.

Future studies should focus on validating this framework in integrated models, identifying reliable biomarkers for patient stratification, and developing tissue-specific, stage-dependent interventions to enable precise and safe clinical translation.
